# Treatment of Infantile Gastric Fever

**Published:** 1845-11

**Authors:** Golding Bird


*Treatment of Infantile Gastric Fever.* By Golding Bird, A.M., M.D.—The origin of gastric fever occurring among children is usually to be ascribed either to unhealthy ingesta or depraved secretions. The pulv. sodæ comp. of Guy's Pharmacopia, in doses of three to eight grains at night, and a full dose of the pulv. rhæi salin. every morning for a week or so, will in most cases be found a very successful treatment. To the latter compound, so well known to the profession for its almost specific power in these affections, Dr. Fordyce accorded this elaborate praise—"Had I been more ambitious of dying a rich man, than of living a useful member of society, the powers of our anti-hectic powder in curing, as if by miracle, the hectic fever and the swelled bellies of children in this town would have remained a secret while I lived."

